# Outcomes of Inguinal Hernia Repair Over Five Years in a Scottish District General Hospital

**DOI:** 10.7759/cureus.103766

**Published:** 2026-02-17

**Authors:** Jayanta Bhowmick, Jeyakumar Apollos

**Affiliations:** 1 General Surgery, Dumfries and Galloway Royal Infirmary, Dumfries, GBR

**Keywords:** district general hospital, inguinal hernia repair, laparoscopic repair, open inguinal hernia repair, recurrence, retrospective audit, surgical outcomes research

## Abstract

Background: Inguinal hernia repair is one of the most frequently performed general surgical procedures worldwide. Recurrence remains a key quality indicator.

Objective: To assess outcomes of inguinal hernia repair in a district general hospital in Scotland, focusing on recurrence rates and describing patterns observed according to surgical approach, surgeon grade, and patient demographics.

Methods: A retrospective descriptive audit was conducted of 900 inguinal hernia repairs performed between 2019 and 2024. Data on hernia type, repair method, complications, and recurrence were extracted from electronic records and analyzed using descriptive statistics.

Results: A total of 900 inguinal hernia repairs were analyzed. The overall recurrence rate was 2.22% (n = 20/900). Laparoscopic repair demonstrated a lower recurrence rate (n = 2/188, 1.08%) compared with open repair (n = 18/712, 2.5%). Higher recurrence rates were observed in patients aged over 60 years (n = 14/20, 70%) and in recurrent hernia repairs (n = 3/62, 4.8%) compared with primary repairs (n = 17/838, 2.02%).

Conclusion: Recurrence rates were comparable to rates reported in the published literature. Surgical approach, patient age, and hernia complexity influenced the outcomes. Ongoing audits and standardized training are essential to maintain quality.

## Introduction

Inguinal hernia repair continues to be one of the most commonly performed surgical procedures worldwide, with over 20 million hernioplasties carried out annually [1]. Despite significant advancements in surgical techniques and materials, hernia recurrence remains an important outcome measure in evaluating the long-term effectiveness of inguinal hernia repair. Recurrence rates are influenced by a range of factors, including the choice of surgical technique, patient-specific comorbidities, and the surgeon’s level of expertise and experience [2]. Historically, tension-based tissue repair techniques such as the Bassini and Shouldice procedures were widely practiced. However, these methods relied on sutured approximation of native tissues under tension and were associated with higher recurrence rates. The introduction of tension-free mesh repair represented a major advancement in inguinal hernia surgery, significantly reducing recurrence rates and improving postoperative outcomes. Currently, mesh-based repair techniques such as the open Lichtenstein procedure and minimally invasive laparoscopic approaches, including totally extraperitoneal (TEP) and transabdominal preperitoneal (TAPP) repairs, are described in international guidelines as the best evidence-based options for primary inguinal hernia repair [3,4].

The management of recurrent inguinal hernias poses additional challenges due to the presence of scar tissue, fibrosis, and altered anatomical planes resulting from previous surgeries, which can complicate dissection and increase the risk of intraoperative complications [5,6]. Evidence suggests that laparoscopic approaches may offer distinct advantages in these cases by providing enhanced visualization and access to the preperitoneal space, thereby reducing tissue trauma and facilitating optimal mesh placement [7]. Moreover, the strategic use of mesh in recurrent hernia repairs, particularly when laparoscopic techniques are employed following primary open mesh repairs, has demonstrated improved long-term outcomes, including lower rates of reoperation and fewer postoperative complications [8]. These findings highlight the importance of individualized surgical planning that carefully considers the patient’s prior surgical history, hernia characteristics, and overall health status to optimize repair durability and functional recovery.

Given these considerations, this study aims to explore the impact of surgical approach, surgeon experience, and patient demographics on recurrence rates, employing a comprehensive retrospective analysis.

## Materials and methods

This retrospective study encompassed adult patients who underwent inguinal hernia repair at a district general hospital, Dumfries and Galloway Royal Infirmary, Dumfries, Scotland, United Kingdom. In the UK healthcare system, a district general hospital provides secondary-level care, delivering routine elective and emergency surgical services. While it supports surgical training and regular teaching activities, it does not function as a tertiary referral center. The study covered a five-year period from January 2019 to January 2024. The study included all adults aged 18 years and older who received either elective or emergency inguinal hernia repair. Inclusion of both elective and emergency cases was intentional to reflect routine surgical practice within a district general hospital. Both primary and recurrent hernias were considered to provide a comprehensive overview of surgical outcomes in this patient population. Data were meticulously extracted from the hospital’s electronic health records, capturing a wide range of variables such as patient demographics; hernia characteristics, including anatomical type (direct, indirect, or pantaloon, as documented in operative records) and laterality; surgical approach and specific techniques employed; surgeon experience, as indicated by grade; anesthesia modality; postoperative complications; and incidence of hernia recurrence. Bilateral hernias were analyzed as two separate repairs. The choice of surgical approach was based on routine clinical practice. Specific indications for approach selection, patient comorbidity profiles, and surgeon preference were not systematically recorded in this retrospective dataset.

This study was conducted as a retrospective descriptive audit. Descriptive statistical methods were applied to analyze the collected data. Recurrence rates were stratified according to surgical approach (open versus laparoscopic), type of hernia repair, and surgeon grade. No inferential statistical testing or multivariable adjustment was performed, and results are presented descriptively. Given the relatively low number of recurrence events, findings are reported as observed patterns within this cohort without implying causal relationships.

Follow-up and definition of recurrence

Follow-up data were obtained through review of electronic health records up to the study end date (January 2024). Hernia recurrence was defined as a clinically documented diagnosis of recurrent inguinal hernia recorded during outpatient review, emergency presentation, cross-sectional imaging, or subsequent operative intervention. Re-operation for recurrence was considered confirmatory evidence but was not the sole criterion for diagnosis.

## Results

Following the outlined methodology, we present the demographic distribution and surgical outcomes of inguinal hernia repairs analyzed.

Demographics

A total of 900 inguinal hernia repairs were performed during the study period. The median age of the cohort was 67 years (range 18-92 years). Patients aged ≥60 years constituted the majority of the study population (n = 597, 66%), followed by those aged 40-59 years (n = 238, 27%) and those aged under 40 years (n = 65, 7%), as summarized in Table 1. The majority of patients were male (n = 853, 95%), while females accounted for 5% of cases (n = 47), as shown in Table 2. Patients aged over 60 years represented the majority of recurrence cases, accounting for 70% of all recurrences (n = 14/20).

**Table 1:** Age distribution

Age Group	Number (n)	Percentage (%)
<40 years	65	7%
40–59 years	238	27%
≥60 years	597	66%

**Table 2:** Demographics (gender)

Gender	Count	Percentage
Male	853	95
Female	47	5

Hernia type and anatomical classification

The majority of hernias were primary inguinal hernias (n = 838, 93%), while recurrent hernias accounted for a smaller proportion (n = 62, 7%), as summarised in Table 3. Anatomically, indirect (lateral) hernias were the most common type (n = 483, 54%), followed by direct (medial) hernias (n = 388, 43%) and pantaloon hernias (n = 29, 3%), as shown in Table 4. Right-sided hernias were slightly more prevalent (n = 497, 55%) compared with left-sided hernias (n = 403, 45%), as outlined in Table 5.

**Table 3:** Hernia type

Type	Count	Percentage
Primary	838	93
Recurrent	62	7

**Table 4:** Anatomical types of inguinal hernia

Type of hernia	Numbers	Percentage
Direct (Medial)	388	43
Indirect (Lateral)	483	54
Pantaloon	29	3

**Table 5:** Anatomical side of inguinal hernia

Side of the hernia	Numbers	Percentage
Right	497	55
Left	403	45

Repair approach

Almost all hernia repairs (n = 898/900, 99.7%) were performed using mesh-based techniques. The Lichtenstein repair was the predominant method among open procedures. Suture (anatomical) repair was performed in two cases (n = 2/712, 0.3%). Open repair was the most commonly employed surgical approach, performed in 79% of cases (n = 712/900), while laparoscopic repair accounted for 21% (n = 188/900), as detailed in Table 6

**Table 6:** Repair approach

Approach	Count	Percentage
Open	712	79
Laparoscopic	188	21

Open Repair Techniques

Among open repairs, the Lichtenstein mesh technique was used in the vast majority of cases (n = 710/712, 99.7%), with suture anatomical repair performed in only two cases (n = 2/712, 0.3%), as shown in Table 7.

**Table 7:** Types of open inguinal hernia repair

Type of open repair	Number	Percentage
Lichtenstein (Mesh)	710	99.7
Suture anatomical repair	2	0.3

Laparoscopic Repair Techniques

Of the laparoscopic repairs, TEP repair was the most frequently performed technique (n = 104/188, 55%), followed by TAPP repair (n = 84/188, 45%), as presented in Table 8.

**Table 8:** Types of laparoscopic groin hernia repair TEP: totally extraperitoneal repair; TAPP: transabdominal preperitoneal repair

Type of laparoscopic repair	Number	Percentage
TEP	104	55
TAPP	84	45

Surgeon grade

Consultant surgeons performed 46% of procedures (n = 417/900), specialty, associate specialist, and specialist (SAS) doctors performed 22% (n = 195/900), and supervised trainees performed 32% (n = 288/900), as summarised in Table 9. 

**Table 9:** Surgeon grade SAS: specialty, associate specialist, and specialist

Surgeon grade	Count	Percentage
Consultant	417	46
SAS doctor	195	22
Trainee	288	32

Type of anesthesia technique

General anaesthesia was used in the majority of cases (n = 877/900, 97.0%), while spinal anaesthesia was used in 2.7% of cases (n = 20/900) and local anaesthesia in 0.3% (n = 3/900), as shown in Table 10.

**Table 10:** Type of anesthesia technique

Type	Count	Percentage
General	877	97.0
Spinal	20	2.7
Local	3	0.3

Recurrence rates

The overall recurrence rate across all procedures was 2.22% (n = 20/900), as shown in Table 11. Recurrence was higher following open repair (n = 18/712, 2.5%) compared with laparoscopic repair (n = 2/188, 1.08%). Recurrent hernia repairs demonstrated a higher recurrence rate (n = 3/62, 4.8%) compared with primary hernia repairs (n = 17/838, 2.02%). The majority of recurrences were identified through clinical assessment during subsequent hospital presentations or outpatient follow-up, with re-operation performed where clinically indicated.

**Table 11:** Recurrence rates

Category	Recurrence rate (%)
Overall	2.22
Primary	2.02
Recurrent	4.8
Open repair	2.5
Laparoscopic repair	1.08

**Figure 1:**
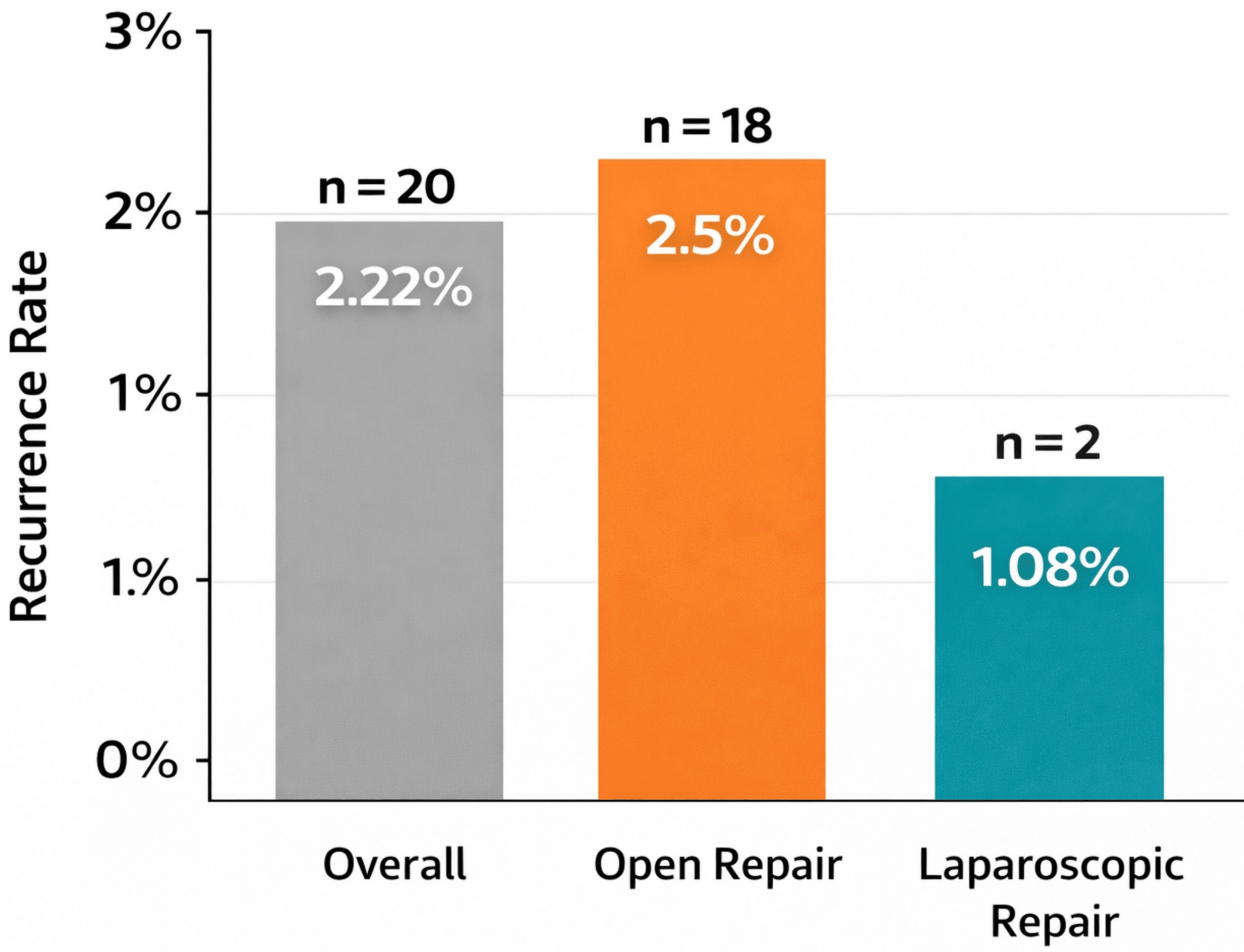
Recurrence rates by type of repair Bar chart comparing recurrence rates among different surgical approaches for inguinal hernia repair. Laparoscopic repair showed the lowest recurrence rate (1.08%) compared to open repair (2.5%) and the overall average (2.22%).

When stratified by surgeon grade, recurrence rates were 2.6% for consultants (n = 11/417), 1.5% for SAS doctors (n = 3/195), and 2.08% for trainees (n = 6/288), as presented in Table 12. Recurrence rates by repair approach are illustrated in Figure 1.

**Table 12:** Recurrence rate by surgeon grade SAS: specialty, associate specialist, and specialist

Surgeon grade	Recurrence rate (%)
Consultant	2.6
SAS doctor	1.5
Trainee	2.08

Postoperative complications

**Figure 2:**
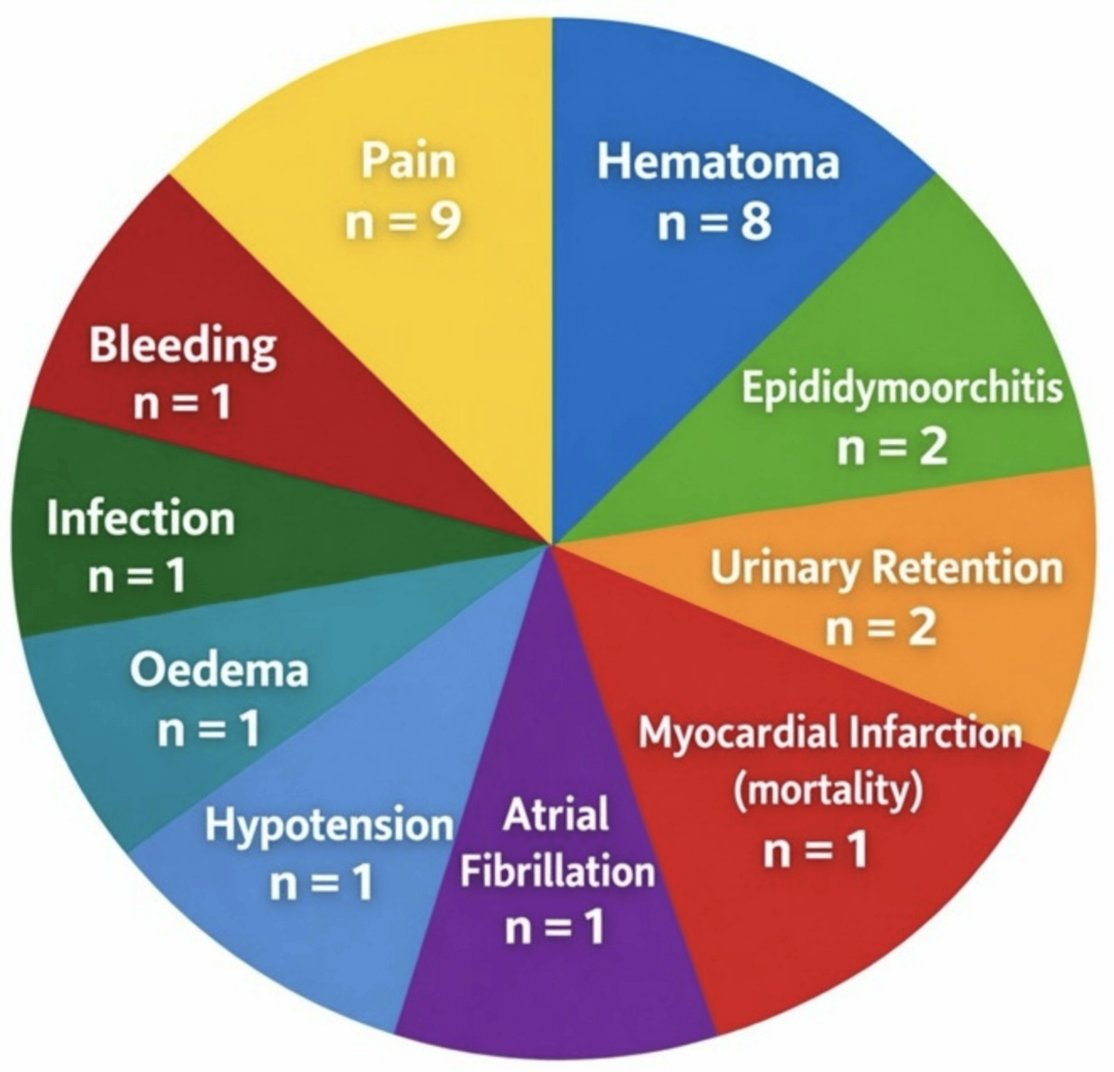
Post-operative complications Distribution of postoperative complications observed in 900 inguinal hernia repairs. Pain and hematoma were the most common complications, followed by bleeding, infection, edema, epididymoorchitis, urinary retention, hypotension, atrial fibrillation, and one case of myocardial infarction.

Postoperative adverse events, including both surgical and perioperative medical complications, occurred in 3.6% of cases (n = 33/900). The most frequently observed complications were postoperative pain (n = 9, 1.0%) and hematoma formation (n = 8, 0.9%). Other complications included bleeding (n = 1, 0.1%), surgical site infection (n = 1, 0.1%), edema (n = 1, 0.1%), epididymoorchitis (n = 2, 0.2%), urinary retention (n = 2, 0.2%), hypotension (n = 1, 0.1%), atrial fibrillation (n = 1, 0.1%), and one case of perioperative myocardial infarction resulting in mortality (n = 1, 0.1%). These findings are summarized in Table 13 and illustrated in Figure 2.

**Table 13:** Types of complications

Complication type	Frequency, n (%)
Pain	9 (1.0%)
Hematoma	8 (0.9%)
Bleeding	1 (0.1%)
Infection	1 (0.1%)
Edema	1 (0.1%)
Epididymoorchitis	2 (0.2%)
Urinary retention	2 (0.2%)
Hypotension	1 (0.1%)
Atrial fibrillation	1 (0.1%)
Myocardial infarction (mortality)	1 (0.1%)

The median length of hospital stay was one day (range 0-21 days). The majority of patients (n = 660, 73%) were discharged on the same day, while 18% (n = 165) stayed for one day and 9% (n = 75) required a hospital stay of two or more days. Length-of-stay data are summarised in Table 14.

**Table 14:** Length of hospital stay

Length of stay (days)	Number (n)	Percentage (%)
0 (same-day discharge)	660	73%
1 (one day)	165	18%
2 or more days	75	9%

**Figure 3:**
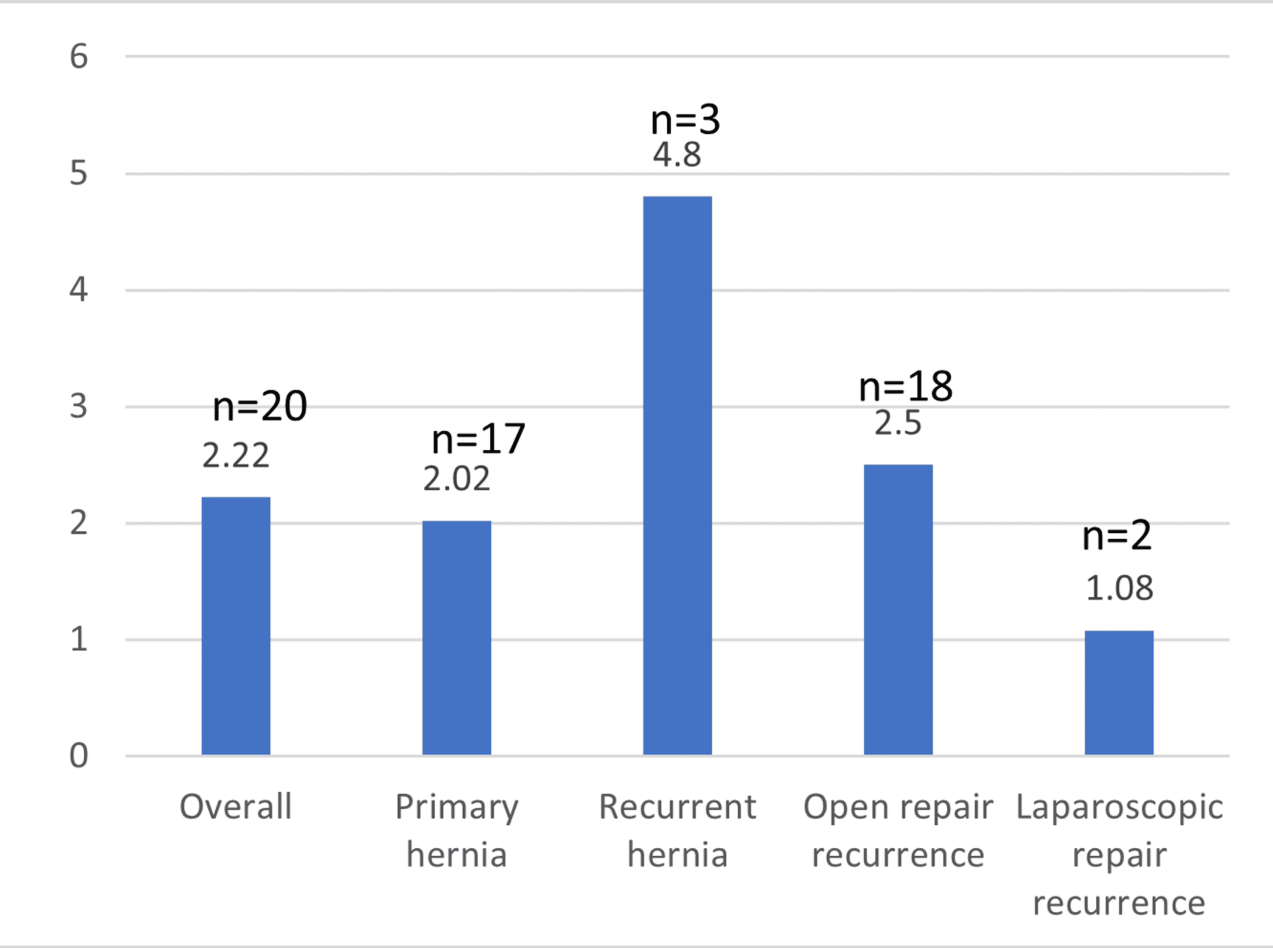
Recurrence rates by category (%)

Recurrence rates across overall cases, primary and recurrent hernias, and by surgical approach are illustrated in Figure 3.

## Discussion

Our study identified an overall recurrence rate of 2.22%, which aligns with the findings of international studies concerning mesh repairs [2,4]. According to Smith et al., the recent figures in the UK are 4% [9]. Laparoscopic repairs showed lower recurrence rates than open techniques, consistent with the findings of Eklund et al. and Bay-Nielsen et al., who advocated minimally invasive approaches in recurrent cases [7,8]. Notably, Bisgaard et al. used reoperation rate as a surrogate for recurrence, which should be interpreted accordingly [8]. Recurrent hernias have a notably higher recurrence rate, emphasizing the complexity of redo operations. Our results echo those of Haapaniemi et al., who reported increased reoperation rates in recurrent cases [6]. Older patients (>60 years) also had a higher recurrence, likely due to tissue degeneration, which aligns with the study conducted by Junge et al. [10]. Emerging evidence suggests that alterations in connective tissue composition and potential genetic predisposition may contribute to hernia formation, although preventive strategies targeting these mechanisms are not yet established and remain an area for further research.

The majority of cases operated on in our series were operated under general anesthesia (97%), and only around 3% were operated under regional or local anesthesia. These findings are similar to those of a previous study [11]. In the UK, inguinal hernia repair may be performed under general, regional (spinal or epidural), or local anesthesia. While spinal and local techniques are supported by evidence in selected patients [12], anesthetic choice in routine practice reflects institutional configuration, theater workflow models, patient factors, and surgeon-anesthetist preference within a district general hospital setting. The predominance of general anesthesia in this cohort represents local service delivery patterns rather than a prescriptive recommendation over regional techniques.

Additionally, we observed a trend toward higher recurrence rates in cases operated on by junior surgeons compared to the senior SAS surgeons. This aligns with findings from other studies, in which surgeon experience has been shown to significantly influence recurrence and complication outcomes. Structured supervision and adherence to standardized operative techniques are essential for reducing recurrence across all surgeon grades [13].

Interestingly, the recurrence rate was not lowest among consultants in this cohort. Consultants demonstrated a recurrence rate of 2.6%, compared with 2.08% for trainees and 1.5% for SAS doctors. These differences should be interpreted cautiously, given the retrospective descriptive design of the study and the absence of objective measures of case complexity. It is possible that consultants were more frequently involved in technically demanding or recurrent cases; however, this study did not collect detailed complexity indicators to formally assess this. Similar observations have been reported in the literature, where higher-risk hernia repairs are associated with increased recurrence rates [14].

In addition to these findings, the existing literature suggests that surgical outcomes are not only a function of technique but also influenced by patient selection, institutional protocols, and the volume of procedures performed. High-volume centers are associated with improved hernia repair outcomes and reduced complications, supporting the centralization of complex cases [15]. The study does not specify exact figures; however, it identifies high-volume centers and Centers of Excellence (COE) as those performing a significantly greater number of hernia surgeries compared to the average. These centers employ surgeons who have surpassed their learning curve, utilize evidence-based clinical treatments, document outcomes in a hernia registry or database, and conduct follow-up comparisons of outcomes with benchmark data to facilitate continual improvement. While high-volume centers have been associated with improved outcomes in various surgical domains, inguinal hernia repair, particularly uncomplicated open repair, is widely performed across different hospital settings with generally favorable results. Centralization may benefit selected complex or recurrent cases; however, routine hernia surgery remains safely deliverable within district general hospitals. Audit and feedback systems, such as those utilized in this study, are important for maintaining quality standards and monitoring outcomes across all levels of surgical practice [6].

About the future of hernia surgery, robotic-assisted hernia repair represents an evolving technique within minimally invasive surgery. Current evidence suggests potential technical advantages; however, its higher costs, longer operative times, and learning curve limit widespread implementation. At present, robotic hernia surgery is primarily utilized within specialized or tertiary centers, and further high-quality studies are required to define its long-term comparative benefits over established open and laparoscopic approaches [16].

Limitations of the study

This study has several limitations inherent to its retrospective design. Although higher recurrence rates were observed among consultant-performed repairs, any interpretation regarding case complexity remains speculative. While certain operative details may have been documented in operative notes, complexity indicators such as defect size, degree of fibrosis, emergency presentation characteristics, or American Society of Anesthesiologists (ASA) grade were not prospectively standardized or systematically extracted for quantitative analysis. Consequently, these factors could not be reliably incorporated into the study’s statistical evaluation.

The inclusion of emergency cases may introduce heterogeneity in patient characteristics and operative complexity. However, emergency repairs constituted a small proportion of the cohort and were included to reflect real-world clinical practice within a district general hospital setting.

Additionally, outcomes were not stratified by comorbidity burden, validated frailty indices, prostatism status, urology referrals, or prophylactic catheterization practices, as these variables were not systematically recorded in the dataset. These factors may influence perioperative outcomes and recurrence risk. As a result, findings should be interpreted descriptively rather than as evidence of causal relationships. These limitations highlight the need for prospective studies incorporating standardized complexity, comorbidity, and risk assessment measures. In addition, focused retrospective analyses specifically designed to capture these parameters may further clarify their impact on outcomes.

Furthermore, recurrence detection in this study was based on clinically documented presentations within available electronic health records. Asymptomatic or subclinical recurrences may not have been captured, potentially leading to underestimation of the true recurrence rate. Follow-up duration was inherently heterogeneous due to the retrospective design, with some patients having longer observation periods than others. This variation may influence the reported recurrence rate, particularly for more recent cases. Finally, as a single-center study conducted within a district general hospital, the findings may not be fully generalizable to other institutions with different patient populations, referral patterns, or service configurations.

## Conclusions

Inguinal hernia repair at our institution achieved recurrence rates consistent with those reported in the literature, supporting the effectiveness of current surgical protocols within a district general hospital setting. Recurrence was higher in recurrent and technically complex hernias, underscoring the challenges associated with redo surgery. Laparoscopic repair demonstrated lower recurrence rates compared with open repair in this cohort.

Continued audit, structured training, and prospective data collection remain essential to maintaining and improving surgical outcomes. Complex or recurrent hernias may benefit from management by surgeons with substantial experience in hernia repair or abdominal wall reconstruction, particularly in technically demanding cases. Ongoing evaluation of service delivery models will help ensure high-quality, evidence-based care for patients undergoing inguinal hernia repair.
